# How and when does proactive personality predict career adaptability? A study of the moderated mediation model

**DOI:** 10.3389/fpsyg.2024.1333829

**Published:** 2024-06-21

**Authors:** Hui Li, Ziyue Xu, Suhao Song, Hui Jin

**Affiliations:** ^1^School of Economics and Management, Nanjing Tech University, Nanjing, China; ^2^School of Economics and Management, Jiangsu University of Science and Technology, Zhenjiang, China

**Keywords:** proactive personality, career adaptability, strengths use, managerial coaching, moderated mediation model

## Abstract

In the present study, we explored the relationship between proactive personality and career adaptability to construct a cross-level moderated mediation model based on the conservation of resources (COR) theory. By conducting a time-lagged study involving three data collection points from 587 employees across 104 teams in China, we examined how and when proactive personality predicts employees' career adaptability using strengths use as a mediator and managerial coaching as boundary conditions. The results revealed that proactive personality predicted strengths use, which, in turn, influenced career adaptability, with managerial coaching moderating the indirect relationship between proactive personality and career adaptability. Consequently, our findings suggest that, in contexts where managerial coaching lacks guidance, facilitation, and inspiration, a proactive personality encourages employees to leverage their strengths, subsequently enhancing their career adaptability. Finally, we discuss the theoretical and practical implications of our findings, address limitations, and propose directions for future research.

## 1 Introduction

In today's diverse and ever-evolving career landscape, an individual's adaptability plays a pivotal role in surmounting career hurdles and setbacks. It significantly influences both satisfaction and performance in an individual's professional journey (Johnston, 2018). In the contemporary job market, an employee's adaptability not only contributes to their competitive edge but also fosters robust social connections within their organization, correlating positively with career success (Wang et al., 2022). This area has garnered considerable attention from scholars (Federici et al., 2021; Kundi et al., 2022).

Embedded within the career construction theory (Savickas and Porfeli, 2012), which conceptualizes career development as driven by adaptation to a social environment with the goal of person–environment integration, career adaptability is recognized as a psychosocial resource essential for navigating transitions, fostering development, and overcoming career-related adversities. Studies have explored its predictive nature and the mechanisms guiding its effectiveness (Hirschi and Valero, 2015). Notably, researchers have identified certain personality traits, such as core self-evaluations (Valls et al., 2020), extraversion (Bakker et al., 2019), self-esteem (Cai et al., 2015), and future temporal focus (Zacher, 2014), as predictors of career adaptability, solidifying the association between personality and adaptability. Moreover, proactive personality has been a focus, with research emphasizing its role in enhancing employee adaptability and development (Tolentino et al., 2014; Jiang, 2017; Green et al., 2020).

Individuals exhibiting high levels of proactive personality tend to actively seek opportunities, take initiative, and drive meaningful change (Bateman and Crant, 1993). They persistently pursue occupational goals and adapt to challenges (Parker et al., 2010). These attributes are regarded as predictors of career adaptability and could enhance employees' careers, fostering a sense of control, curiosity, concern, and confidence in career-related endeavors. Notably, career adaptability demands proactive behavior tailored toward achieving career objectives (Savickas and Porfeli, 2012). However, a previous meta-analysis study (e.g., Rudolph et al., 2017) provides strong evidence that proactive personality exhibits only a modest relationship (β = 0.30) with career adaptability. Furthermore, there remains a gap in the literature on career management in understanding how and when proactive employees align their traits to enhance career adaptability.

Highly proactive individuals tend to gather information from various sources and process it meticulously (Parker et al., 2010), influencing their attitudes and behaviors positively toward their career development. Recent studies have explored psychological and behavioral variables, including self-efficacy in career decisions (Hou et al., 2014), career exploration (Cai et al., 2015), thriving at work (Jiang, 2017), career planning (Valls et al., 2020), perceived social support, and specialty identity (Hu et al., 2021), as mediators in this process. However, these studies are yet to explore the specific proactive work behaviors contributing to enhancing career adaptability. Hence, there is a need to explore the behavioral mechanism centered around proactive work behaviors such as strengths use.

The strengths-based approach, which has attracted the attention of massive research bodies on human strengths and virtues (Peterson and Seligman, 2004), defines strengths use as work behaviors that maximize effectiveness and foster career development (Littman-Ovadia et al., 2014). This approach has been found to increase work authenticity, engagement (Bakker and van Wingerden, 2021), and overall career-related well-being (Matsuo, 2020), making it increasingly relevant in contemporary workplaces as well as a growing focus in positive organizational behavior research. Despite this significance, few studies have investigated the predictors of strengths use from a personality perspective (e.g., Kong and Ho, 2016; van Woerkom et al., 2016c). According to the conservation of resources (COR) theory (Hobfoll, 1989), people tend to control and retain resources that they value. People are intrinsically motivated to acquire, use, and invest their resources, and a proactive orientation serves as a critical personal resource, aiding individuals in acquiring career resources that facilitate learning, growth, and interconnectedness (Demerouti et al., 2001). Therefore, understanding how a proactive personality translates into effective career adaptability may hinge on comprehending strengths use.

Additionally, the interactionist perspective on career adaptability suggests that adaptability arises from a blend of personality traits, the career context, and their interplay (Savickas and Porfeli, 2012). A previous research study has demonstrated that situational factors can influence personal traits that, in turn, are linked to behaviors, and contextual cues can either bolster or diminish the connection between proactive personality and career behaviors (McCormick et al., 2019). Numerous studies have highlighted leadership as a pivotal contextual factor for influencing employee career behavior (Hou et al., 2014; Chen et al., 2021). While existing research has examined how personal and situational factors interact to impact individual career adaptability, limited attention has been paid to specific local contexts characterized by guidance, facilitation, and inspiration, such as managerial coaching (Heslin et al., 2006), and how it shapes the relationship between individuals' traits and career adaptability. Therefore, this study explores managerial coaching as a boundary condition that influences the indirect effects of proactive personality on career adaptability through strengths use.

The current study aims to explore the mediating effect of strengths use and the moderating effect of managerial coaching on the relationships between proactive personality, strengths use, and career adaptability. First, drawing from the COR theory, we examine whether a proactive personality is linked to career adaptability and investigate the mediating effect of strengths use. Second, we integrate mediation and cross-level moderation effects to explore how managerial coaching moderates these relationships. This study holds two theoretical implications for the literature on strengths use and career adaptability. On the one hand, it investigates how individual personality traits differentiate career adaptability through specific work behaviors (i.e., strengths use), drawing from the intrinsic motivational state perspective (Kong and Ho, 2016). On the other hand, it delves into situational factors, such as managerial coaching, suggesting that employees are differently influenced in their ability to utilize their strengths to enhance career adaptability, and this finding is consistent with previous research that situational cues can influence the proactive personality–career adaptability relationship (Zacher, 2014).

## 2 Theoretical backgrounds and hypotheses

### 2.1 Proactive personality and career adaptability

Proactive personality is characterized by individuals possessing an attitudinal and behavioral inclination to identify and overcome environmental obstacles, thereby bringing about changes in their circumstances (Bateman and Crant, 1993). Proactive employees actively seek job and organizational information and make significant changes to bolster job performance, driven by the motivation to learn and enhance their capabilities (Parker et al., 2010). Several studies have revealed a positive correlation between proactive personality and various career-related outcomes such as career success (Seibert et al., 1999), career competencies (Plomp et al., 2016), career adaptability (Jiang, 2017), and professional success (Chen et al., 2021). Moreover, in the context of contemporary boundaryless careers (Arthur, 2014), where employees are encouraged to self-manage their careers, personality traits play a critical role in career development and growth because individuals who have greater initial resources are more likely to experience resource gain.

Proactive individuals tend to take proactive steps in career development, seeking feedback and transitioning occupationally, which aids in creating career networks, managing challenges, and adapting to changes. Given that career adaptability is a multifaceted construct, research studies have developed a higher-order construct to measure and assess it, encompassing aspects such as concern, control, curiosity, and confidence (Savickas and Porfeli, 2012). Concern involves looking ahead and seeking new vocational tasks; control entails taking individual responsibility for one's own actions; curiosity involves exploring possible selves; and confidence represents the belief in overcoming obstacles to achieve career goals. Employees with high levels of proactivity tend to be more active than passive, thereby bringing about positive changes in their environment, and are relatively less constrained by situational forces compared to employees with lower levels of proactivity (Bateman and Crant, 1993).

For instance, a meta-analysis conducted by Fuller and Marler (2009) revealed that possessing a proactive disposition is linked to career success. This success is often measured through job performance, which reflects contest mobility, and through taking charge or voice behavior, indicating proactive mobility. In general, personality significantly impacts career success, as it fundamentally influences behavioral choices (Seibert et al., 1999). Proactive personality proves beneficial for employability by enhancing career self-efficacy (Tolentino et al., 2014). Moreover, previous studies have consistently found a positive association between proactivity and all facets of career adaptability (Zacher, 2014; Hirschi et al., 2015).

According to the COR theory (Hobfoll, 1989), individuals are intrinsically motivated to acquire, use, and invest their resources. Those possessing greater personal resources are more likely to secure additional resources. Proactive employees inherently possess dispositional resources that enable them to pursue their career goals actively. This proactive nature contributes to an increase in career adaptability resources. In essence, proactive individuals exhibit high motivation to shape favorable career environments and demonstrate determination in understanding their role in their career trajectory by nurturing curiosity, confidence, control, and concern. Previous studies have demonstrated that proactive individuals possess high intrinsic motivation and increased self-efficacy, leading to enhanced career adaptability (Jiang, 2017). Building upon these findings, this study proposes the following hypothesis:

**Hypothesis 1:** Proactive personality positively correlates with career adaptability.

### 2.2 The mediating role of strengths use

Strengths are considered innate attributes that encompass an individual's talents, capabilities, and social intelligence, shaping their potential to pursue valued outcomes (Peterson and Seligman, 2004). Theoretical perspectives on positive psychology have emphasized both possessing and using strengths. The strengths-based approach was based on the belief that employees are most successful at achieving their goals when they can identify and utilize their strengths. Despite psychologists and practitioners making significant progress in identifying individual strengths through analysis, there has been limited research focused on utilizing these strengths. Strengths use refers to an individual's unique traits and abilities contributing to their exceptional performance (Wood et al., 2011).

Kong and Ho (2016) identified that leader autonomy support, intrinsic motivation, and independent self-construal promote employees' use of strengths. Moreover, Bakker and Van Woerkom (2018) highlighted four critical predictors of strengths use: personal initiative, job autonomy, organizational support, and personal development opportunities. Additionally, studies have shown that perceived organizational support for strengths use and personal initiative significantly contribute to leveraging strengths (van Woerkom et al., 2016a). Proactive personality, considered a key precursor of strengths use, represents one of the proactive behaviors (Crant, 2000). Consequently, personality, as an abstract disposition, can manifest through strengths use (Ding and Lin, 2020), guiding job crafting toward leveraging individual strengths (Kooij et al., 2017).

As indicated by previous studies, proactive employees are more inclined to utilize their strengths (Chen et al., 2021), aligning with their development needs and preferences, thereby making their careers more congruent with their self-concept, abilities, and aspirations. Drawing from the COR theory (Hobfoll, 1989), individuals who utilize their strengths effectively can adopt more promotive coping strategies, thus replenishing more resources. Therefore, this study posits that highly proactive individuals are more likely to utilize their strengths to align with their personality traits and cater to their preferences, leading to our second hypothesis.

**Hypothesis 2:** Proactive personality positively correlates with strengths use.

As previously mentioned, the character strengths theory suggests that employing one's strengths positively impacts their wellbeing. Strengths use enables individuals to operate at their personal best, providing authenticity and energy (Wood et al., 2011). When individuals capitalize on their strengths, they tend to be more focused on their tasks, increasing their likelihood of success. Numerous studies have shown a positive correlation between strengths use and wellbeing indicators, such as happiness, vitality, and life satisfaction (e.g., Forest et al., 2012; Bakker et al., 2019), as well as between strengths use and increases in self-esteem (Wood et al., 2011), work engagement (Bakker and van Wingerden, 2021), and self-rated performance (van Woerkom et al., 2016b). Recent research also associates strengths use with assertiveness, self-efficacy, and resilience (Bakker and van Wingerden, 2021), all of which contribute to career advancement. Hence, it is proposed that strengths use positively impacts career concern, control, confidence, and curiosity, thereby influencing career adaptability.

Jiang (2017) discovered that perceived internal status acts as a motivational mechanism linking proactive orientation to career outcomes, demonstrating that meaningful work positively relates to performance through strengths use. Expanding on the COR theory, the gain spiral of resources posits that individuals perceive resources as valuable and engage in adaptive behaviors, with coping strategies mediating between personal resources and career-related outcomes (Hobfoll, 2001). Accordingly, a proactive personality could foster proactive behavior, such as strengths use, to attain adaptive resources. Highly proactive employees are better positioned to invest in (strengths use) and acquire additional resources (career adaptability). Previous studies have shown the role of strengths use as a mediator between autonomy support and helping behaviors (Kong and Ho, 2016). Hence, it is predicted that highly proactive individuals utilize their strengths to achieve heightened levels of career adaptability. As factors other than strengths use might mediate the associations between a proactive orientation and career-related outcomes, we posit our next hypothesis:

**Hypothesis 3:** Proactive personality has a positive indirect effect on career adaptability via strengths use.

### 2.3 Moderating role of managerial coaching

*Managerial coaching* involves a leader or supervisor facilitating learning among subordinates to acquire and apply knowledge and capabilities through directed behaviors (Ellinger et al., 2003). This coaching aids employees in solving problems more efficiently or performing tasks through guided discussions and activities (Hagen, 2012). It illuminates work-related goals and pathways, enhancing subordinates' workplace wellbeing (Zhao and Liu, 2020). Managerial coaching acts as a contextual factor aiding employees in maximizing their personal career potential (Segers and Inceoglu, 2012). A previous research study has indicated the benefits of managerial coaching on work-related outcomes such as learning (Matsuo, 2020), in-role behavior (Tanskanen et al., 2019), and job satisfaction (Kim and Egan, 2013). Managerial coaching directly influences the career outcomes of employees (e.g., occupational commitment, Kuo et al., 2014; proactive career behavior, Huang and Hsieh, 2015) by removing barriers related to career development.

Unlike stable traits such as personal characteristics, strengths use is motivated by personality traits, the environment, and their interplay (Bakker and van Wingerden, 2021). Recent findings by Ding and Lin (2021) revealed that individual-focused transformational leadership and core self-evaluation interact to influence strengths use through positive affect. These effects might align with managerial coaching, providing guidance, suggestions, and inspiration to help subordinates realize their potential (Heslin et al., 2006). The COR theory also suggests that one resource can be substituted by another, generating equivalent value (Hobfoll, 2001), and supportive context can be a kind of compensation that contributes to the maintenance of resource reservoirs and resource gains. When managerial coaching exhibits more empowerment and facilitation behaviors (Ellinger et al., 2011), even less proactive employees may feel inspired and empowered, resulting in increased strengths use. However, highly proactive employees primarily respond to leadership through prescribed change, future-oriented initiatives, and self-initiative, rather than focusing on managerial coaching skills. Consequently, strengths use by highly proactive employees depends more on their personality and less on their leaders' actual behaviors.

In addition to the COR theory, McCormick et al. (2019) conducted a field survey from the perspective of person–situation interaction (Mischel, 1977), since both leadership and personality are capable of triggering the follower's behavior, and found that transformational leadership moderated the relationship between employee proactive orientation and proactive behavior. Recently, Bakker and van Wingerden (2021) discovered that the combination of organizational and individual approaches toward strengths use significantly accounted for variance in work engagement. Kim and Kuo (2015) highlighted managerial coaching as a manager-initiated support for subordinates' career developments. We propose that managerial coaching influences the connection between proactive personality and strengths use, suggesting that individuals perceiving higher coaching may rely less on their proactive traits.

As previously mentioned, we proposed that a proactive personality tends to enhance employees' use of strengths, subsequently fostering their career adaptability. When managerial coaching is low, employees are more likely to depend on a proactive personality to obtain resources, thereby satisfying their innate needs for utilizing strengths. Consequently, enhanced use of strengths is more likely to elevate the level of career adaptability (Plomp et al., 2016). Conversely, in teams with higher managerial coaching, employees may rely less on personal traits as their psychological needs might be met through managerial coaching. Consequently, their use of strengths, potentially less influenced by proactive personality, might not significantly increase career adaptability, as observed in teams with lower managerial coaching. Therefore, we posit that individuals receiving lower managerial coaching are more prone to be influenced by personality traits to sustain the use of strengths, consequently feeling more adapted in their careers. Considering the multi-faceted nature of our sample, we propose managerial coaching as a contextual cue. Based on a multilevel model, we address the cross-level moderated mediation effect on career adaptability. Specifically, managerial coaching attenuates the indirect effect of followers' proactive orientation on career adaptability through strengths use, leading to our fourth hypothesis:

**Hypothesis 4:** Managerial coaching moderates the indirect effect of a proactive personality on career adaptability through strengths use, weakening the relationship for individuals with higher levels of managerial coaching.

**Figure 1:**
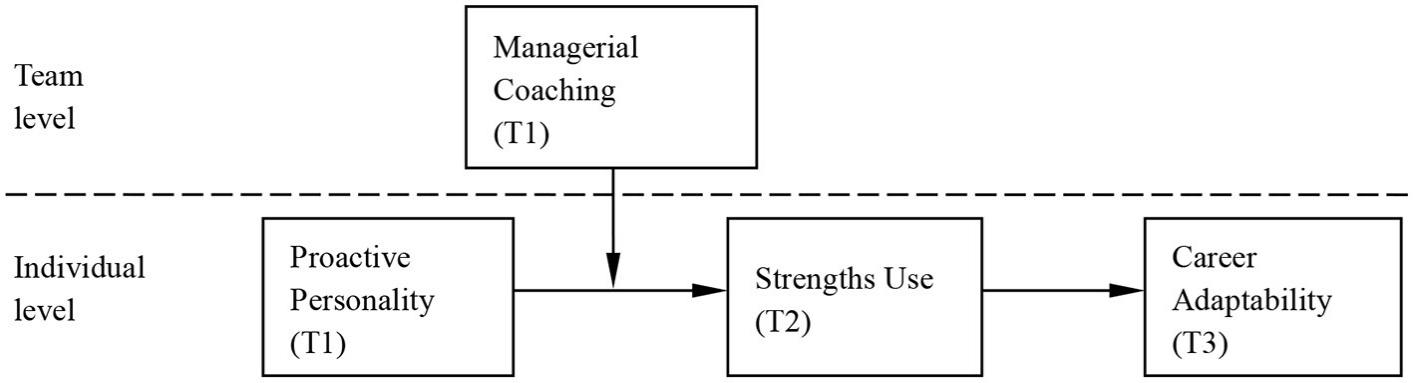
The hypothesized moderated mediation model.

To summarize, the hypothesized model of our study is illustrated in Figure 1.

## 3 Methods

### 3.1 Participants and procedure

The data for this study were collected from 12 hotel service companies in East China, involving full-time employees. Questionnaires were delivered in a hardcopy format with the assistance of the human resources management (HRM) department. At Time 1, 720 questionnaires were distributed to rate proactive personality, managerial coaching, and controlled variables. A total of 642 responses were received from 118 teams, accounting for an 89.2% response rate. Approximately after 2 months (Time 2), questionnaires were used to report strengths use. Among the respondents from the first phase, 614 participants from 112 teams returned to the second survey, resulting in a 95.6% response rate. After 4 months (Time 3), questionnaires assessing career adaptability were distributed to those who completed the second survey. A total of 595 participants completed the third set of questionnaires, accounting for a 96.9% response rate. Match codes were used across the three surveys to ensure the confidentiality of the identity of participants. After eliminating incomplete and unmatched questionnaires, the final sample comprised 587 responses from 104 teams. To encourage participation, financial incentives were provided at each phase of data collection.

The total sample consisted of 587 employees from 104 teams. Among the employees, 32.2% were male participants. Regarding the educational qualification of the participants, 16.9% held a high school degree, 42.3% held a partial college degree, 35.9% held a bachelor's degree, and 4.9% held a master's degree. In terms of age distribution, 43.3% were younger than 30 years, while 39.5% were aged between 30 and 40 years. Regarding tenure, 22.8% had a tenure below 3 years, while 54.9% had a tenure between 3 and 5 years. In terms of team size, 34.6% belonged to teams comprising fewer than five people, 51.9% belonged to teams comprising 5–9 people, and 13.5% belonged to teams comprising more than 15 people. We did not find a significant difference between our final sample (*n* = 587) and the initial sample (*n* = 642) in terms of demographics or any of the studied variables.

### 3.2 Measures

By following Brislin's (1986) translation–back translation procedure, the initial English versions of the measures were translated into Chinese. Two bilingual graduate students were tasked with the translation, and subsequently, the measures were translated back by them. The authors, along with two bilingual professors specializing in organizational behavior, compared the original measures with the back-translated versions for the final Chinese survey. The participants rated the survey items on a seven-point Likert scale.

#### 3.2.1 Proactive personality

This study utilized a 10-item version developed by Seibert et al. (1999) to assess proactive personality. An example statement was “I excel at identifying opportunities.” The Cronbach's α coefficient was 0.88, and the composite reliability was 0.91.

#### 3.2.2 Strengths use

Strengths use was measured using a five-item scale developed by van Woerkom et al. (2016b). A sample statement was “I use my talents at work.” The Cronbach's α coefficient was 0.89, and the composite reliability was 0.93.

#### 3.2.3 Managerial coaching

The study employed the 10-item coaching behavior instrument developed by Heslin et al. (2006) to evaluate managerial coaching. Items included “My manager helps me to examine my performance” (Guidance), “My manager encourages me to explore and seek out new alternatives” (Facilitation), and “My manager encourages me to continuously develop and improve” (Inspiration). The Cronbach's α coefficient for the general managerial coaching factor was 0.81, and the composite reliability was 0.87.

#### 3.2.4 Career adaptability

Savickas and Porfeli's (2012) Career Adapt-Abilities Scale, comprising 24 items, was used to assess career adaptability. Items included “Preparing for the future” (Concern), “Sticking up for my beliefs” (Control), “Probing deeply into questions I have” (Curiosity), and “Learning new skills” (Confidence). The Cronbach's α coefficient for the general adaptability factor was 0.85, and the composite reliability was 0.87.

*Control variable*: Previous research showed that employees' demographic characteristics (Zacher, 2014; Rudolph et al., 2017) can influence their career adaptability, both of which were controlled for in the analysis. Gender was coded as 0 for “women” and 1 for “men.” Education was coded as 1 for a “high school degree,” 2 for “partial college degree,” 3 for a “bachelor's degree,” and 4 for a “master's degree.” Team size was coded as 1 for “below five people,” 2 for “5–9 people,” 3 for “10–15 people,” and 4 for “above 15 people.” Age and dyad tenure were self-reported in years.

### 3.3 Data aggregation

The statistical analysis was conducted using Mplus 8.0 (Muthén and Muthén, 2017) to evaluate the convergent and discriminant validity of the study variables and test hypotheses. The intra-class correlation coefficients (Bliese, 2000) for managerial coaching were examined first, revealing ICC_1_ = 0.24 and ICC_2_ = 0.73, indicating that 24% of the variance in managerial coaching was found between teams. Additionally, the average interrater agreement (R_wg_) across groups for managerial coaching was 0.86, suggesting appropriate within-team agreement (James, 1982). These findings suggest that managerial coaching could be aggregated to a higher level.

## 4 Results

Confirmatory factor analysis (CFA) was employed to assess the discriminant validity of the study variables, considering the data collected from three surveys to minimize the potential for common method bias (Podsakoff et al., 2003). The data indicated that the hypothesized four-factor model, namely, career adaptability, strengths use, managerial coaching, and proactive personality, demonstrated an acceptable fit (χ^2^*/df* = 1.683; CFI = 0.925; TLI = 0.918; RMSEA = 0.056), outperforming other models. Table 1 presents the results of the CFA.

**Table 1:** The results of confirmatory factor analysis for the measures of variables studied.

**Model**	**χ^2^**	**df**	**χ^2^/df**	**CFI**	**TLI**	**RMSEA**
Four-factor model	378.675	225	1.683	0.925	0.918	0.056
Three-factor model: PP and SU combined	564.984	228	2.478	0.876	0.841	0.079
Two-factor model: MC and other factors	749.108	230	3.257	0.823	0.797	0.095
Two-factor model: CA and other factors	796.027	230	3.461	0.735	0.704	0.112
One-factor model	1,204.434	231	5.214	0.628	0.539	0.138

PP, proactive personality; SU, strengths use; MC, managerial coaching; CA, career adaptability.

The means, standard deviations, and correlations among the study variables are detailed in Table 2. The correlations align with predictions; notably, career adaptability significantly relates to proactive personality (*r* = 0.46, *p* < 0.01), strengths use (*r* = 0.51, *p* < 0.01), and managerial coaching (*r* = 0.32, *p* < 0.01). Proactive personality is significantly related to strengths use (*r* = 0.54, *p* < 0.01), while the correlation between proactive personality and managerial coaching is non-significant.

**Table 2:** Means, standard deviations, and correlations among variables studied.

**Variables**	**Means**	**SD**	**1**	**2**	**3**	**4**
1. Proactive personality	5.31	0.18	***0.82***			
2. Strengths use	5.12	0.12	0.54^**^	***0.67***		
3. Managerial coaching	5.54	0.28	0.28	0.36^*^	***0.74***	
4. Career adaptability	5.26	0.34	0.46^**^	0.51^**^	0.32^**^	***0.69***

N = 587.

^*^p < 0.05, ^**^p < 0.01.

Bold-faced numerals on the diagonal represent the square root of the average variance extracted.

Table 3 presents the outcomes of our data analyses. Hypothesis 1 exhibited a positive correlation between proactive personality and career adaptability. Model 6 revealed a significant correlation between proactive personality and career adaptability (γ = 0.47, *p* < 0.01), aligning with our expectations and supporting Hypothesis 1. Hypothesis 2 suggested that a proactive personality positively predicts strengths use. As demonstrated in Model 2, the findings indicated a positive association between proactive personality and strengths use (γ = 0.56, *p* < 0.01), confirming Hypothesis 2 as anticipated. Hypothesis 3 proposed a mediated pathway from proactive personality to career adaptability through strengths use. In Model 7, strengths use was introduced after Model 6. The results consistently displayed a significant coefficient of proactive personality on career adaptability; however, the effect size attenuated (γ = 0.23, *p* < 0.01). These findings suggest that strengths use partially mediates the relationship between proactive personality and career adaptability. Notably, an indirect effect of proactive personality on career adaptability through strengths use was observed: *b* = 0.26. By using 5,000 resamples for 95% Monte Carlo confidence intervals (Muthén and Muthén, 2017), the analysis confirmed the significance of the indirect effect with a 95% CI = [0.201, 0.319], in line with our expectations and supporting Hypothesis 3.

**Table 3:** The results of hierarchical linear modeling.

**Variables**	**Strengths use**				**Career adaptability**				
	**M1**	**M2**	**M3**	**M4**	**M5**	**M6**	**M7**	**M8**	**M9**
Intercepts	5.27^**^	5.25^**^	5.21^**^	5.18^**^	3.86^**^	3.87^**^	3.85^**^	3.79^**^	3.92^**^
**Level-1 variables**									
Age	0.11	0.11	0.12	0.11	0.08	0.08	0.08	0.09	0.07
Education	0.14	0.16	0.15	0.15	0.04	0.05	0.05	0.04	0.05
Gender	0.07	0.07	0.08	0.09	0.12	0.13	0.13	0.12	0.13
Dyad tenure	0.10	0.11	0.09	0.09	0.06	0.06	0.05	0.05	0.04
Proactive personality		0.56^**^	0.55^**^	0.52^**^		0.47^**^	0.23^**^	0.24^**^	0.23^*^
Strengths use							0.52^**^	0.51^**^	0.52^*^
**Level-2 variables**									
Team size	0.08	0.08	0.07	0.10	0.07	0.07	0.07	0.07	0.08
Managerial coaching			0.36^**^	0.21^**^				0.22^**^	0.14
**Cross-level interaction**									
Proactive personality × managerial coaching				−0.24^**^					0.09
Strengths use × managerial coaching									−0.12
*R^2^*	0.06	0.18	0.25	0.36	0.05	0.18	0.29	0.38	0.43
*ΔR^2^*		0.12	0.07	0.11		0.13	0.11	0.09	0.05

^*^p < 0.05, ^**^p < 0.01.

**Figure 2:**
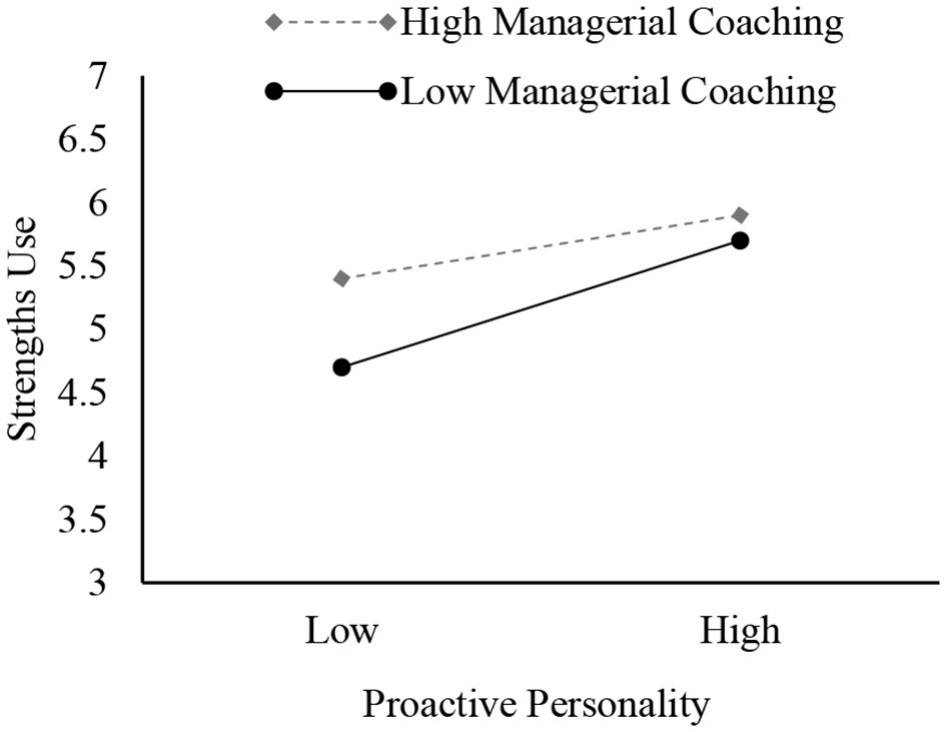
The moderation effect of managerial coaching on the relationship between proactive personality and strengths use.

Hypothesis 4 proposed that managerial coaching would moderate the indirect associations between proactive personality and career adaptability mediated by strengths use. To investigate the cross-level interplay and integrate the lower-level mediation pathway for analysis (Bauer et al., 2006), this study estimated the direct moderated effect of managerial coaching and the mediation effect of a proactive personality on career adaptability contingent upon varying degrees of managerial coaching. In Model 4 and Model 9, the first-stage moderated effect of managerial coaching was significant (proactive personality-strengths use, γ = −0.24, *p* < 0.01), while the second-stage moderated effect (strengths use-career adaptability, γ = −0.12, *p* > 0.05) and the direct moderated effect (proactive personality–career adaptability, γ = 0.09, *p* > 0.05) were not significant. The conditional mediation effect (Preacher et al., 2007) demonstrated significance at 1 standard deviation below the mean (M-1SD; *b* = 0.398, *t* = 5.96, *p* < 0.01), whereas it was non-significant at 1 standard deviation above the mean (M+1SD; *b* = 0.174, *t* = 0.83, *p* = 0.54). Figure 2 illustrates the cross-level interaction effects with simple slopes.

Table 4 presents the results of the moderated mediation effects. The indirect relationships between proactive orientation through strengths use and career adaptability significantly differed at both levels of managerial coaching. Specifically, the indirect effect was stronger [effect = 0.15, SE = 0.03, 95% BCCI = [0.024, 0.279]] when managerial coaching was low (i.e., M −1 SD) and weaker [effect = 0.06, SE = 0.04, 95% BCCI = [−0.023, 0.096]] when managerial coaching was high (i.e., M +1 SD). This highlights that a proactive personality indirectly impacts career adaptability through strengths use, especially when managerial coaching is low, thereby reinforcing Hypothesis 4.

**Table 4:** The conditional indirect(s) effects of proactive personality on career adaptability at varying levels of managerial coaching.

**Moderator**	**Stage**	**Effect**
	**First**	**Second**	**Direct**	**Indirect**	**Total**
High managerial coaching	0.21	0.26^**^	0.32	0.06	0.38^*^
Low managerial coaching	0.47^**^	0.25^**^	0.27^*^	0.15^**^	0.42^*^
Difference	0.26^**^	0.01	0.05	0.09^**^	0.04^*^

^*^p < 0.05, ^**^p < 0.01.

## 5 Discussion

The current study delved into the process mechanisms and boundary conditions shaping the influence of a proactive personality on career adaptability. Our findings revealed a significant correlation between proactive personality and strengths use, where the latter partially mediated the relationship between an employee's proactive orientation and career adaptability. Moreover, in our moderated mediation analysis, we observed that, when managerial coaching levels were higher, the associations between proactive personality, strengths use, and career adaptability were lower. This study highlights managerial coaching as a boundary condition influencing how proactive personality translates into strengths use and, consequently, career adaptability.

### 5.1 Theoretical implications

By developing a cross-level moderated mediation model, this study significantly contributes to understanding how and when an employee's proactive orientation relates to their career adaptability. It should be noted that our findings extend the literature on career adaptability by proposing strengths use as a specific form of proactive work behavior within the framework of the COR theory. While previous research has explored how proactive orientation leads to various career outcomes such as career planning (Valls et al., 2020), trustworthiness, and psychological empowerment (Huang and Hsieh, 2015), our study emphasizes that highly proactive employees tend to exhibit more strengths use behavior, thereby enhancing their career adaptability.

This study identifies strengths use as a behavioral mechanism that elucidates the effects of a proactive personality on career adaptability, thereby bridging the gap between personality traits and career-related outcomes. Our results complement the literature by establishing a trajectory of strengths use in career management, elucidating a pathway through which a proactive personality influences career adaptability (Hirschi et al., 2015; Hu et al., 2021). Additionally, this is the first study to integrate strengths use and leadership perspectives, shedding light on their interactive effects on career-related outcomes. Drawing from an interactionist perspective and career constructive theory, we demonstrate how the benefits of personality for individual career development might hinge on relational resources, aligning with the views of Delle and Searle (2022). This study highlights that strengths use mediates the associations between employee proactive orientation and career adaptability, emphasizing that managerial coaching, as a crucial situational cue, might weaken these relationships.

Our findings address Savickas and Porfeli's (2012) call for further exploration of the moderating role of managerial coaching in understanding how a proactive personality impacts career adaptability in different contexts. This study operationalizes career adaptability as a psychosocial resource, broadening the exploration of the study of career adaptability beyond adaptivity (Tolentino et al., 2014). It emphasizes the importance of an individual's work context in understanding their career adaptability. By illustrating that managerial coaching could mitigate these relationships, our study highlights that relational resources could substitute for personal resources, catalyzing proactive behavior.

### 5.2 Practical implications

The results of our study yield significant practical implications. First, our findings highlight the importance of a proactive orientation as a pivotal factor in evaluating qualified candidates for career adaptability in human resource management. Organizations seeking to enhance their competitive edge should consider employing and retaining highly proactive employees. Emphasizing the utilization of strengths not only enables individuals to tailor their career development but also highlights the potential for managers to adopt effective coaching skills to bolster their subordinates' career adaptability.

Second, focusing our attention toward strengths use opens up a new avenue for maximizing staff potential, and leveraging strengths use can potentiate the role of career adaptive resources in promoting positive career outcomes. Managers should assist subordinates in identifying, honing, and effectively employing their strengths, fostering authenticity, particularly in today's dynamic and demanding career landscapes. Aligning work with employees' strengths can significantly enhance and sustain career adaptability.

Third, while a proactive personality fosters strengths use in situations with lower managerial coaching, its impact on strengths use decreases in environments with higher managerial coaching. Nonetheless, Figure 2 indicates that the mean strengths use in high managerial coaching situations surpasses that of low managerial coaching, regardless of employees' differences in proactivity. Therefore, cultivating employees' strengths use and career adaptability involves training managers in coaching skills, engaging in coaching activities (Bakker and Van Woerkom, 2018), and providing career-oriented training and self-development opportunities (Bakker and van Wingerden, 2021) to employees.

### 5.3 Limitations and future research

Our study has some limitations to be noted. First, relying solely on self-ratings (Savickas and Porfeli, 2012) for survey data might not fully capture the true variance reflecting employee career adaptability. Future research should incorporate multiple raters or evaluate career adaptability from supervisors' perspectives. Second, while statistical evidence supports our study's hypothesis, alternative explanations might exist, such as the possibility that employees with high adaptability are more inclined to use their strengths (Klehe et al., 2021). It seems likely that strengths use, on the one hand, and career adaptability, on the other hand, are inversely related, thereby leading to a positive gain spiral. Future research should focus on investigating whether career adaptability mediates the relationship between proactive personality and strengths use.

Additionally, exploring organizational factors, such as human resource management, as potential moderators between employees' proactive orientation and career adaptability could be valuable. This study mainly focused on strengths use on an individual level; however, examining how employees interact to identify, cultivate, and utilize strengths at a team level could be insightful (van Woerkom et al., 2016b). Finally, while our research centered on career adaptability in Chinese organizations, exploring these findings in other cultural contexts would contribute to generalizing the study's results (Savickas and Porfeli, 2012). Different countries may have diverse demands and influences on career adaptability, thus warranting broader cross-cultural examinations.

## 6 Conclusion

This study integrated perspectives on proactive personality, strengths use, and managerial coaching to elucidate the mechanisms influencing career adaptability. It highlighted a proactive personality as pivotal in acquiring, safeguarding, and cultivating resources to enhance career adaptability. Individuals with a proactive personality demonstrate heightened career adaptability, partly due to their ability to tailor their skills by leveraging their strengths. Consequently, strengths use acts as a conduit, fostering skill development and enhancing motivation, thereby facilitating career adaptability among proactive employees. Additionally, the indirect impact of an employee's proactive personality on career adaptability through strengths use is contingent upon varying levels of managerial coaching. Thus, this study highlights the interconnected nature of behavioral mechanisms, such as strengths use, and contextual factors, such as managerial coaching, in shaping an individual's career adaptability.

## Data availability statement

The original contributions presented in the study are included in the article/supplementary material, further inquiries can be directed to the corresponding author.

## Ethics statement

The studies involving humans were approved by the ethical standards of the Nanjing Tech University and with the 1964 Helsinki Declaration and its later amendments or comparable ethical standards. The studies were conducted in accordance with the local legislation and institutional requirements. The participants provided their written informed consent to participate in this study.

## Author contributions

HL: Conceptualization, Methodology, Project administration, Writing – original draft, Writing – review & editing, Funding acquisition, Investigation, Validation. ZX: Data curation, Formal analysis, Resources, Software, Supervision, Visualization, Writing – original draft. SS: Conceptualization, Formal analysis, Methodology, Resources, Software, Validation, Writing – original draft. HJ: Data curation, Funding acquisition, Investigation, Project administration, Supervision, Visualization, Writing – original draft.
